# Asymptomatic Dandy-Walker syndrome in an adult

**DOI:** 10.11604/pamj.2014.19.15.3974

**Published:** 2014-09-08

**Authors:** Hatim Belfquih, Brahim Elmostarchid

**Affiliations:** 1 Department of Neurosurgery, Mohammed V Military Teaching Hospital, Rabat, Morocco

**Keywords:** Dandy-Walker malformation, agenesis of vermis, cyst of posterior fossa

## Image in medicine

This 34 years old man presented with moderate and persistent headache occurring 48 hours following Benin head injury. There were no symptoms of increased intracranial pressure. Neurological examination was unremarkable without cerebellar ataxia or psychomotor retardation .the patient was submitted to CT scan and magnetic resonance imaging (MRI) of the brain that revealed agenesis of cerebellar vermis and large posterior fossa cyst communicating with the enlarged fourth ventricle suggestive of Dandy-Walker malformation without hydrocephalus. He was managed conservatively and he is doing well following 2 years ago. The Dandy-Walker syndrome (DWS) is a rare posterior fossa malformation and more rarely observed in adults. This case is unique in that the patient has been entirely asymptomatic with this abnormality since birth. The preserved cortical cytoarchitecture and the rarity of additional neurodevelopmental changes in DWS adults may explain the mild or absence of clinical expression, compared with DWS infants.

**Figure 1:**
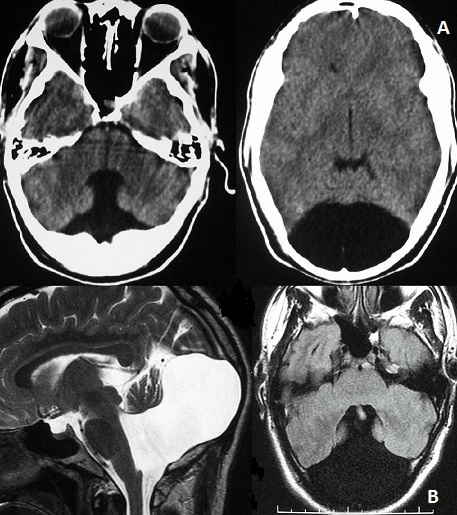
(A) CT scan showing agenesis of cerebellar vermis, large posterior fossa cyst communicating with the fourth ventricle suggestive of Dandy-Walker malformation (left) and thinning of the occipital bone (right). (B): magnetic resonance imaging, T2 sagittal view (left) and T2 Flair axial view (right), showing agenesis of cerebellar vermis and large cerebrospinal-fluid filled cyst in the posterior fossa that is confluent with the fourth ventricle without hydrocephalus

